# Building a Robust A-P Axis

**DOI:** 10.2174/138920212800793348

**Published:** 2012-06

**Authors:** Alysha Heimberg, Edwina McGlinn

**Affiliations:** EMBL Australia, Australian Regenerative Medicine Institute, Monash University, Wellington Road, Clayton, 3800, Australia

**Keywords:** Hox gene, microRNA, A-P patterning, miR-10, miR-196.

## Abstract

Since the last common ancestor of Metazoa, animals have evolved complex body plans with specialized cells and spatial organization of tissues and organs. Arguably, one of the most significant innovations during animal evolutionary history was the establishment of a bilateral plane of symmetry on which morphological features (e.g. tissues, organs, appendages, skeleton) could be given specific coordinates within the animal along the anterior-posterior (A-P) and dorsal-ventral (D-V) axes. Hox genes are a known group of eumetazoan transcription factors central to regulating A-P patterning, but less well known and under current investigation is the broader regulatory landscape incorporating these genes, including microRNA (miRNA) regulation. The degree to which evolutionarily conserved targeting of Hox genes by Hox-embedded miRNAs contributes directly to A-P patterning is under investigation, yielding contrasting information dependent on the organism and miRNA of interest. The widespread A-P patterning defects observed in recent *miR-196* loss-of-function studies solidifies the importance of miRNA regulation in Hox genetic hierarchies, and elucidating the developmental and evolutionary importance of all Hox-embedded miRNAs remains a challenge for the future.

## EVOLUTION OF THE ANTERIOR-POSTERIOR AXIS AND HOX GENES

The anterior-posterior (AP) and dorsal-ventral (DV) polar axes establish the bilateral symmetrical body plan. Bilateral symmetry is thought to have evolved between 800 and 700 million years ago in the last common ancestor of cnidarians (e.g. jellyfish) and eumetazoa (e.g. humans), as it is lacking in the sponge, an asymmetrical animal that likely resembles the last common ancestor of Metazoa [1,2]. Coincident with establishing bilateral symmetry was the acquisition of a conserved set of genes called the Hox genes [3]. Hox genes were discovered in the fruit fly *D. melanogaster* as master regulators of developmental patterning along the A-P axis [4,5]. Since this time, orthologous Hox complexes have been discovered throughout bilaterians indicating a deep origin for the molecular networks driving A-P patterning [reviewed in 6]. 

**Fig. (1):**
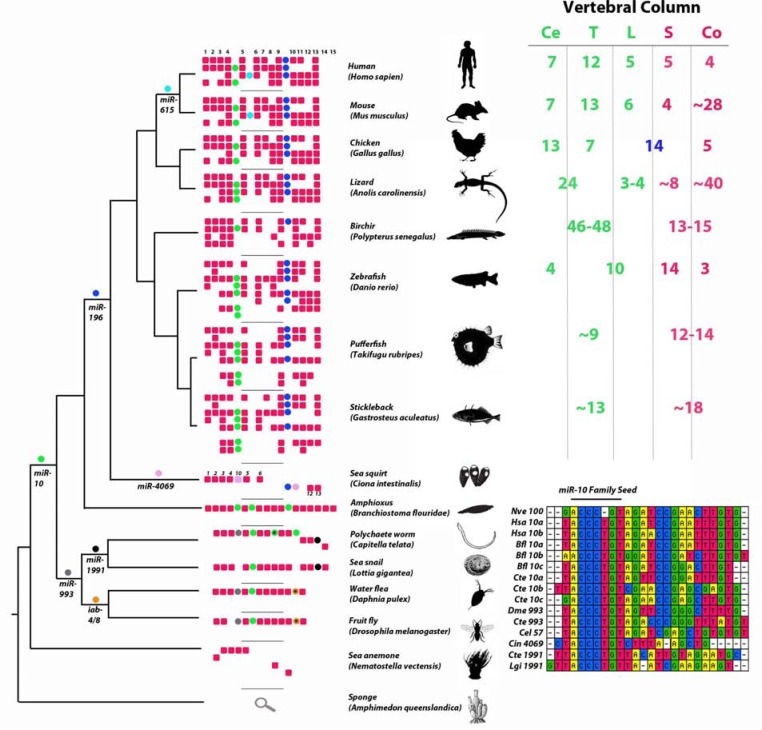
**The Hox clusters among animals.** Red boxes are Hox genes and colored circles are microRNA genes, *miR-10* (green), *miR-196* (dark blue), *miR-615* (light blue), *miR-993* (gray), *miR-1991* (black), *miR-4069* (pink), *miR-1732* (brown) and *iab-4/8* (orange). Genes clustered together in the genome are shown within the same row for a given Hox cluster. For example, human has four Hox clusters, amphioxus has a single Hox cluster and the sea anemone has seven Hox genes among four genomic scaffolds. Relationships among taxa are represented by the cladogram on the left. Variation in the number of vertebrae of the vertebral column is presented on the right. Ce (cervical), T (Thoracic), L (Lumbar), S (Sacral), and Co (Coccyx). Ce, T, and L are pre-caudal regions and colored green. S and Co are caudal regions and colored red. Note that neither Hox genes nor miRNA genes alone define the length or number of segments within the vertebral column. Alignment of *miR-10* family Hox-embedded microRNAs (lower right). *miR-100* from *Nematostella vectensis* is included in this aligment although it is not a Hox-embedded miRNA but a representative of the most deeply conserved member of the *miR-10* family. *Branchiostoma floridae (Bfl), Caenorhabtitis elegans (Cel), Ciona intestinalis (Cin), Capitella teleta (Cte), Drosophila melanogaster (Dme) Homo sapiens (Hsa), Lottia gigantea (Lgi), and Nematostella vectensis (Nve)* [83-90].

The A-P axis consists of individual segments, some of which are considered serial homologues, such as the repeating segments of the vertebrae or multiple sets of paired appendages in some animals. Serial homologues have diverged over time, and the acquisition of unique fates could be achieved by altering, either subtly or dramatically, an inherently modular developmental gene network. The variation in Hox gene repertoires between species may account for the diversity in segment identity and the micro- and macro-evolution of animal body plans. Certainly with increased Hox complexity came more elaborate segmental identity along the A-P axis between species, but also gradations within segments of a given species. However, morphologically diverse clades of animals (such as human-mouse-chicken) share identical Hox repertoires (Fig. **1**), suggesting that diversity is also achieved by different Hox gene regulation. The major regions of a developing embryo (e.g. cervical-thoracic-lumbar-sacral (C-T-L-S) of the vertebrate axial skeleton) are outlined by the expression of specific Hox genes, and in some cases, differences in Hox expression boundaries are in register with morphological regions. For example, *Hoxa5 *expression extends across the C-T transition in species with markedly different axial formulae such as mouse, chick and alligator, although there are numerous examples of lineage specific differences in Hox expression patterns [7,8]. Understanding the complex regulatory landscape of Hox clusters is fundamental to understanding the building of the A-P body axis. There are multiple transcriptional and post-transcriptional regulatory mechanisms acting on the Hox clusters [reviewed in 9;10-iewed in 9;10-13]. Recently, several microRNA genes have been identified within the Hox clusters (Fig. **1**), and have been shown to target certain Hox genes [14-20].

## HOX GENE EXPRESSION ALONG THE A-P AXIS

Many invertebrate bilaterians have a single cluster of 8-9 Hox genes, although there are gains and losses of Hox genes throughout invertebrate taxa as well as genomic rearrangements (as seen with *Drosophila* and *Ciona*; Fig. **1**). Additional Hox genes were acquired early in chordate evolution, as seen in the cephalochordate amphioxus with 15 clustered Hox genes [21]. Most vertebrates have four Hox clusters as a result of two whole genome duplications events in their last common ancestor, with each cluster retaining a subset of the ancestral chordate Hox genes [22]. Teleost fish underwent a third whole genome duplication and have seven Hox clusters (having lost one), and in zebrafish the eighth cluster is reduced to a single miRNA gene (Fig. **1**) [23,24]. 

While not essential, this genomic clustering facilitates coordinated expression and functionality along the main body axis [reviewed in 25]. During embryogenesis, progressive activation of gene expression from the 3’ to the 5’ end of a Hox cluster results in a correspondingly ordered expression profile from the anterior (head) to the most posterior region (tail). The correlation between genomic position and spatio-temporal gene expression is termed “colinearity” and is a widely conserved feature of the Hox complexes [26,27; discussed in the accompanying review by Durston and colleagues]. Even when cluster context has disintegrated however, restricted A-P expression can still persist [24,28]. 

Clear and reproducible morphological transitions are central to A-P axis formation and are molecularly complex. Very early observations indicated that where co-expressed, posterior Hox genes are dominant over more anterior Hox genes, establishing a hierarchical framework termed phenotypic suppression or posterior prevalence [27,29]. For example, ectopic Ubx (a posterior Hox gene) in the more anterior wing segments gives rise to halteres. Posterior prevalence is a general property that appears to be conserved among triploblastic bilaterians; however, invertebrates and vertebrates exhibit some deviation from this strict hierarchy. In vertebrates, the additional Hox clusters give rise to overlapping and therefore combinatorial Hox expression, giving regions along the AP axis a particular “Hox code” that specifies morphology rather than an strict posterior Hox dominance. This Hox code has been shown to be both quantitative and qualitative in vertebrates and understanding the mechanisms to set a given Hox code is crucial to our understanding of animal development.

## EVOLUTIONARY POSITIONING OF HOX EMBEDDED miRNAs

Aside from the Hox genes themselves, the Hox clusters also include at least eight microRNA genes, *miR-10*, *miR-196*, *miR-615*, *miR-993*, *miR-4069, miR-1991*, *miR-1732* and *miR-iab-4/8* (Fig. **1**; *C. intestinalis *and* C. teleta *hand queried, alignment information available upon request) [17,19,30-33]. These miRNAs have quite diverse evolutionary histories, ranging from the very deep conservation of *miR-10* to more recent acquisitions such as *miR-615* (Fig. **1**). The positioning of these miRNAs within Hox clusters is intriguing from an evolutionary perspective (see below) as well as from a developmental perspective regarding target preference (discussed in subsequent sections).

*miR-10* is conserved among protostomes and deuterostomes (fly and human) although the *miR-10* family is conserved among eumetazoans with *Nematostella vectensis *having a single copy of *mir-100* (3 nt difference from most mature *miR-10* sequences; Fig. **1**) [34]. Importantly, three other Hox embedded miRNAs belong to the *miR-10* family. These include *miR-993* whose position within the Hox cluster is only conserved among protostomes, *miR-1991* identified in lophotrochozoa and *miR-4069* which is unique to *Ciona intestinalis*. *miR-4069*, *miR-1991* and *Capitella miR-10b *may exhibit seed shifting which has been documented for various *miR-10 *orthologs (Fig. **1**) [34,35]. 

*miR-10* resides exclusively between Hox4 and Hox5 in vertebrates and orthologous Hox genes in many invertebrates; however, *miR-10* has additional Hox loci in some species. In amphioxus, which has a single tightly clustered Hox locus likely resembling the ancestral chordate cluster given its full complement of Hox genes, *miR-10* is duplicated twice and these are positioned between Hox5/6 and Hox9/10. Similar duplication and positioning is also now seen in *Capitella teleta*, a protostome, suggesting the intriguing possibility that there is a requirement for a specific miRNA:Hox cluster ratio (dosage) across Bilateria. Interestingly, *C. elegans* has *miR-10* family members not associated with Hox genes but which nonetheless have been shown to target Hox orthologues, such as *miR-57* targeting of *nob-1*, an Abd homologue [36]. 

It is quite striking that multiple Hox-embedded miRNAs have independently evolved within the same syntenic position. *miR-615* is conserved among eutherian mammals (mouse and human, excluding monotremes and marsupials) and is located within the intron of Hoxc5. *miR-196* is conserved among olfactores (*Ciona* and human); in vertebrates *miR-196* is located between Hox9/10 and in *Ciona*, both *miR-196* and *miR-4069* are located upstream of posterior Hox orthologues. In chick, the recently acquired *miR-1732* is positioned adjacent to *miR-196b* in the HoxA cluster. *miR-iab-4/miR-iab-8 *are conserved among arthropods and are located between *abd-A *(Hox8) and *Abd-B* (Hox9-13 orthologue) supporting the idea that positioning of Hox-embedded miRNAs is not random (Fig. **1**). 

### miRNA Biogenesis and Role in Development

**Fig. (2):**
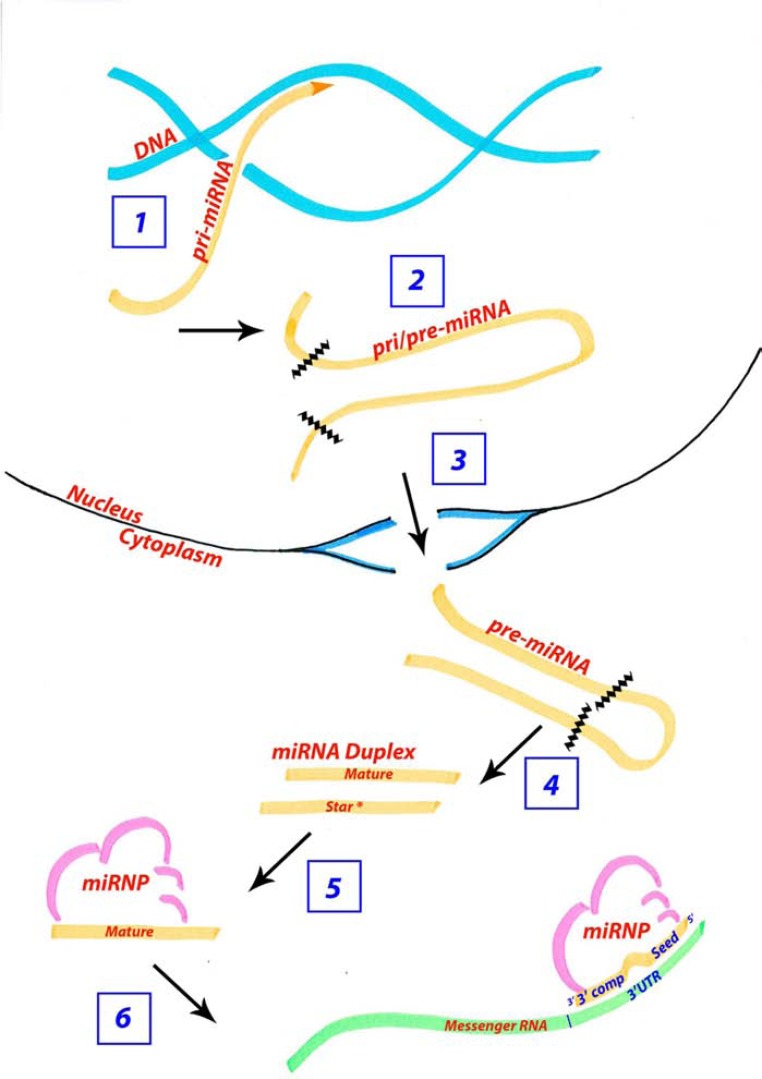
**The microRNA biogenesis pathway is under regulation to modulate the available mature microRNA concentration.** Aside from microRNAs regulating target mRNA transcripts, microRNAs are themselves regulated at several steps during their biogenesis. Steps under regulation include 1) the initial transcription of the primary miRNA, 2) the trimming of the pri-miRNA to the ~70 nucleotide premiRNA, 3) the export of the pre-miRNA from the nucleus to the cytoplasm, 4) the removal of the loop of the hairpin structure giving rise to an ~22 nt duplex, 5) selection of the mature miRNA from the duplex for association with the miRNP and 6) the concentration of miRNP available for targeting.

microRNAs are a class of non-protein-encoding genes that regulate messenger RNA (mRNA) levels and translation [reviewed in 37]. miRNAs are transcribed either under the control of their own promoter or the promoter of a host gene The primary-miRNA (pri-miRNA) transcript forms a stable hairpin secondary-structure and is cleaved once in the nucleus to an ~70 nucleotide (nt) long precursor-miRNA (pre-miRNA) hairpin (Fig. **2**). The pre-miRNA is cleaved a second time in the cytoplasm, to remove the loop of the hairpin and yields an ~22 basepair duplex. One half of this duplex called the “mature” miRNA combines with the rest of the miRNA ribonucleoprotein complex (miRNP) to target an mRNA with a specific target sequence usually in their 3’ untranslated region (UTR). The other half of the duplex called the “star” miRNA is usually degraded although some miRNAs utilize this star sequence to target a second suite of genes (Fig. **2**) [38]. The half-life of a mature microRNA can be as long as 12 days, as with *miR-208*, while the pri- and pre-miRNA appear to have much shorter half-lives [39]. 

Target sequences within an mRNA typically have a 6-7 nt motif that is the reverse complement to nucleotide positions 2-8 of the mature miRNA, termed the “seed” sequence. Additional complementarity may accompany this binding at the seed position. miRNAs that have complementary binding sites spanning the full length of the mature sequence (~22nt) typically result in degradation of the mRNA, while miRNAs with less complementarity affect mRNA stability and translational efficiency [reviewed in 38]. 

Both developing and adult organs have specific spatio-temporal miRNA expression profiles suggesting that the levels of microRNAs are tightly regulated within an animal and that these expression profiles are required for tissue identity [40,41]. Indeed, many pathological phenotypes correlate with abnormal mature miRNA levels. The level of mature microRNA available for mRNA targeting is modulated transcriptionally as well as post-transcriptionally at any of the steps during the biogenesis pathway (Fig. **2**) [42-45]. Importantly, the expression patterns of the pri-, pre-, and mature miRNA have been shown to differ spatially and temporally. Experiments either knocking down or over expressing components of the miRNA biogenesis pathway affect the mature and pre-miRNA products separately and differently depending on the miRNA. Specifically, the levels of mature and pre-*miR-196b* are two to three fold different when altering components driving the pri- to pre- transition, demonstrating that the levels of pre- to mature are not a simple one to one ratio [44]. As well, in an extensive survey of microRNA processing across multiple tissues and cell types, the pre-miRNA was more broadly expressed than the mature-miRNA, with *miR-196* being among the assayed miRNAs in this study [46]. The difference in pri-, pre-, and mature miRNA levels can be visualized by *in situ* hybridization, although the difference in probe design confounds direct comparisons of the levels of each product [47]. 

From an evolutionary perspective, miRNAs are unique in that they are continually acquired through evolutionary time and are among the most highly conserved genes in the animal genome [34]. There are relatively rare instances of secondary loss of miRNAs and phylogenetic relationships among animals can be defined by their miRNA gene repertoires [48-50]. Furthermore, the evolutionary acquisition of novel microRNA families correlates with morphological innovations and the miRNA expression profiles in homologous organs are conserved in many cases [40,41], e.g. the triploblastic bilaterian origin of *miR-1* has conserved expressed in muscle tissue and the vertebrate origin of *miR-122* has conserved expression in the liver. More generally, bursts of microRNA gene acquisition coincide with extensive elaborations of the animal body plan throughout evolutionary history [51,52].

Functional studies ablating mature miRNA biogenesis in zebrafish (maternal-zygotic (M-Z) Dicer loss-of-function) however yielded a surprisingly well-formed embryo at least until mid-embryogenesis [53]. Similar mature miRNA ablation in mouse highlighted an earlier requirement for miRNAs in this species [54], however individual miRNA knockouts generated thus far in mouse [reviewed in 55], rarely cause dramatic phenotypes. These data were initially bewildering given the strong selective pressure to retain these genes throughout evolution. In this light, hypotheses were proposed describing miRNAs as genetic rheostats that act to reduce transcriptional noise. The effect of knocking out a microRNA may not be seen in a single generation unless challenged extrinsically to generate more transcriptional noise, which can not be buffered in the absence of miRNAs. In fact, this hypothesis has been supported experimentally [56]. Additionally, numerous miRNAs have been shown to reinforce commitment to a specific cell lineage by effectively silencing transcript(s) which characterize an earlier or unwanted developmental program [57-59]. In each scenario, miRNA action has been shown impart robustness upon phenotypes and ensure the fidelity of developmental plans. 

### Hox Regulation by microRNAs

**Fig. (3):**
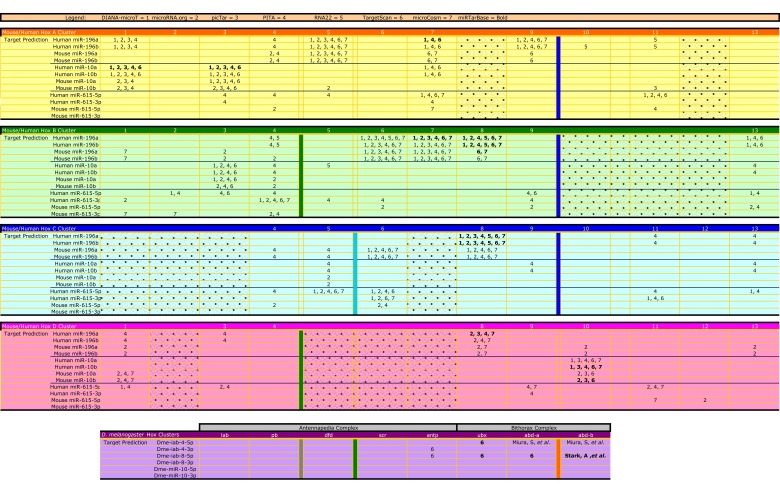
**Summary of predicted Hox gene targets for *miR-10*, *miR-196*, **miR-615 and *miR-iab-4/8*.** Hox genes are predicted targets of Hox-associated miRNAs using several different prediction programs as shown here for the mouse, human, and fly. Each prediction program is given a numerical representative: DIANA-microT (1), microRNA.org (2), PicTar (3), PITA (4), RNA22 (5), TargetScan (6), and microcosm (7). These numbers are listed where they predict a given Hox:miR target interaction. For example, the HoxA cluster has 11 Hox genes and PITA (4) predicts Hoxa4 as a *miR-196* target in humans and mouse. Shaded boxes denote genes that do not exist in that cluster (for example Hoxa8). The HoxA cluster is yellow, B cluster is green, C cluster is blue, D cluster is pink, and the D. melanogaster Hox complexes are purple. The positions of miRNA genes are marked by colored columns, green (*miR-10*), dark blue (*miR-196*), light blue (*miR-615*), orange (*miR-iab 4/8*), and gray (*miR-993*). Note that RNA22 was not queried for mouse targets and the fly predictions are directly from the published literature and/or TargetScan Fly. Although *miR-993* is a Hox-associated miRNA in the fly, there are no known predictions for this miRNA. Targets with experimental support available in miRTarBase (http://mirtarbase.mbc.nctu.edu.tw/) are in bold. [62,88,91-98].

Extensive targeting of Hox genes by Hox-embedded miRNAs has been predicted, though often with minimal agreement between the various prediction programs. Here we provide an updated target analysis (Fig. **3**), incorporating information for *miR-615*, and highlighting those targets with the strongest *in silico* support. Within the human and mouse Hox clusters, *miR-10*, *miR-196*, and *miR-615* have conserved predicted targets in 18 of the 39 Hox 3’ UTRs, or non-conserved predicted targets in ~31 Hox 3’ UTRs. Hox2 and Hox12, of which there are only 2 paralogues of each, have the least computational support as microRNA targets in humans. Additional genes lacking support are Hox4, 11, and 13 in the mouse. Aside from these Hox genes, the remaining 8-11 genes have at least one paralogue as a strongly supported target of a Hox associated miRNA (*miR-10*, *miR*-*196*, or *miR-615*). However, it is important to note that some of the target prediction algorithms are missing 3’ UTR data for some Hox genes biasing the results (see Fig. **3** for details). Also, prediction algorithms search for potential target sites in the 3’ UTR and there is experimental evidence showing that microRNAs can target a mRNA outside of the 3’ UTR [60]. In fact, one example shows *miR-196* binding in the open reading frame of IRGM, an interaction whose misregulation may underly susceptibility to Crohn’s disease [61].

For at least vertebrate miRNAs *miR-10* and *miR-196* as well as *Drosophila miR-iab-4/8*, there appears to be a non-random positioning of Hox-embedded miRNAs relative to their predicted targets; the miRNA is located adjacent to but more 5’ than its Hox targets (see Fig. **3**) [18,20,62]. In the case of *miR-10* (positioned between Hox4 and 5), there is preferential targeting of the Hox1-3 genes, known to pattern the hindbrain and its derivatives. For *miR-196* (positioned between Hox9 and 10), targets cluster within the Hox4-8 genes, know to pattern cervical and thoracic regions. This biased targeting suggests an important role for each miRNA within a localized A-P region, and their posterior positioning relative to targets may suggest a role in refining anatomical transitions. More specifically, since current data indicates Hox-embedded miRNA expressions are broadly consistent with colinearity [14,15,17,18,24,63,64], and they preferentially down regulate anterior Hox genes, miRNA regulation could be invoked to reinforce clearance of anterior developmental programs at posterior locations [20]. Experimental evidence supports this view at least molecularly [14,18,63,65], and an additional role in regulating Hox expression within their endogenous domain has also been identified [16]. 

### Hox miRNA-Target Interactions *In Vivo*

In attempting to define how Hox-embedded miRNAs interact with their Hox targets (eg. switch, failsafe, fine-tuning; see [37] for description of target interactions), a cellular understanding of co-expression is needed which, at least in vertebrates, is yet to be achieved. In addition, the biogenesis of miRNAs is regulated and therefore is not simply on or off within the cell. The biogenesis pathway appears to respond to cellular cues to proceed, pause, or hault the production of mature miRNAs. By doing so, more or less target can be expressed for variable durations and locations. As well, the effective concentration of mature miRNAs can be altered by the presence of targets “absorbing” more or less of the mature transcripts [42,44]. Future investigations into the mechanism of microRNA regulation will hopefully describe these regulatory networks

#### Drosophila miR-10 and miR-iab-4/8

Drosophila *miR-10* is expressed at early developmental stages in what will form central segments, and later within the nerve cord and mid/hindgut [66,67]. *miR-10* is predicted to target many Hox genes, located genomically and spatially both anterior and posterior to the miRNA however to date, no information exists on *in vivo *target regulation during early development. 

The miRNAs *miR-iab-4* and *miR-iab-8* are generated from opposite DNA strands and exhibit mutually exclusive expression domains, *miR-iab-8 *being more posteriorly expressed [63,64,68,69]. Extensive targeting of more anterior Hox genes has been supported with *in vitro* or *in vivo* sensor assays, however to date, endogenous Hox protein regulation has only been demonstrated for Ubx, whose 3’ UTR contains seven potential binding sites [17,63]. At late gastrula stage, *Ubx *expression in the central nervous system is not uniform across individual abdominal segments, and at this site, *miR-iab-4* and *Ubx *expression is largely reciprocal [17]. It is unlikely that the observed anti-correlation is primarily driven by miRNA function [63], however, it is important to note that following deletion of both *miR-iab-4/8*, a more uniform pattern of Ubx protein within individual abdominal segments was detected (*miR-iab-4*+ domain), in addition to a expansion of the posterior boundary of *Ubx* (*miR-iab-8*+ domain). This supports a switch or fail-safe interaction within these individual cells, however the extent to which *miR-iab-4/8 *dampens Ubx is dramatically less than the action of posterior Hox proteins Abd-A and Abd-B [63]. 

### Vertebrate* miR-10 *and *miR-196*

Given the overlapping nature of vertebrate Hox genes [reviewed in 70], it is likely that the primary transcript of Hox associated miRNAs will be co-expressed with at least a subset of its targets. However, there are many layers of regulation in miRNA biogenesis (Fig. **2**), and whether the mature miRNA coincides with all predicted anterior targets is not known. Vertebrate Hox genes usually exhibit strongest expression at their anterior boundary and thus the genomic positioning of *miR-10* or *miR-196* more posterior than their targets suggests that if co-expressed, the miRNA may well be strongest where its target levels are declining. However, it is important to recognize that while these miRNAs will likely be colinear within a given Hox cluster, different clusters have quite different spatio-temporal kinetics. Consistent with this, *miR-196* paralogs themselves exhibit clearly different temporal kinetics in the early mouse [71] and zebrafish [72] embryo. It is quite plausible in fact that a miRNA from one cluster may be temporally activated coincident with targets from another cluster, and thus there is a need to understand each individual miR-target interaction, particularly over developmental time.

Murine *miR-10* is maternally contributed [73], and at later stages of development, *miR-10* paralogs across vertebrates are expressed in a similar manner to Hox4 paralogs [15,18,24,74,75] with which they are often co-transcribed [18,76]. Forced *miR-10* expression in zebrafish can suppress multiple Hox1-3 targets *in vivo*, and does so synergistically with Hoxb4 protein function suggesting a combinatorial approach in limiting anterior Hox gene activity (posterior prevalence) [18]. Complementary *miR-10 *morpholino experiments support this idea and identify a potential function for *miR-10* in clearing “noisy” transcripts that are generated as a result of the compact nature of Hox cluster and therefore close proximity of promoters/enhancers [18]. 

**Fig. (4):**
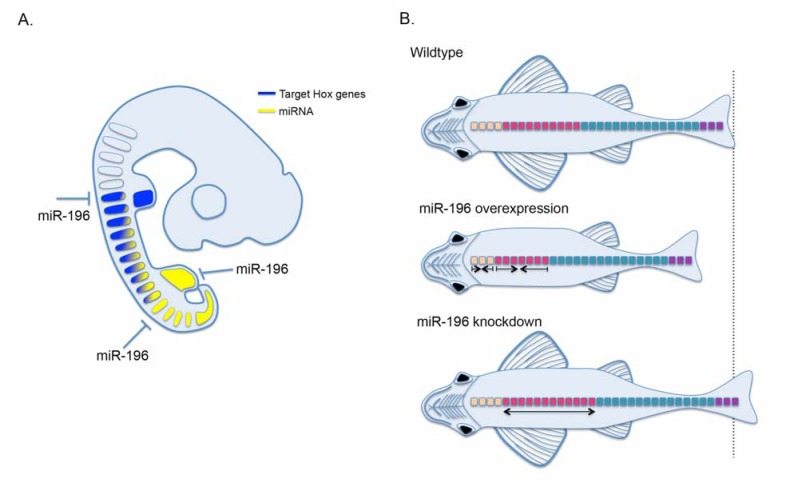
miR-196 regulates Hox gene expression and patterning of the A-P axis. A. *miR-196* (yellow) regulates Hox target expression (blue; *Hoxb8*) both within and at the posterior boundary of its endogenous expression. Note: the regulation of *Hoxb8* at more anterior locations may not be at the embryonic stage depicted, but rather in precursor cells of this region at early stages of development. B. Summary of *miR-196* overexpression and morpholino knockdown results in zebrafish. Relative to wild type, miR-196 overexpression or knockdown regulates pharyngeal arch number as well and the number of pre-caudal rib bearing vertebrae. In addition, *miR-196* overexpression results in complete pectoral fin loss. Vertebral regions are color coded, weberian apparatus (orange), rib-bearing precaudal (pink), caudal (teal) and tail (purple) vertebrae.

*miR-196* has been identified in the 4-8 cell stage in mouse [73] and is maternally contributed in zebrafish [72]. At later stages of development, *miR-196* is broadly expressed in the posterior embryo consistent with its genomic location [14,15,75], and individual paralog expression is beginning to be distinguished [47]. *miR-196* is unique amongst animal miRNAs in that it binds with extensive complementarity to the 3’ UTR of *Hoxb8* in mouse and human, resulting in RNAi like degradation *in vivo *[15,19]. In developing embryos, *miR-196* acts to modulate *Hoxb8* levels both within [16] and immediately posterior to [14] its endogenous expression domain in the paraxial mesoderm, and the detection of Hoxb8 endonucleolytic cleavage products in posterior regions where *Hoxb8 *expression is not observed (eg. hindlimb; [65]) supports a fail-safe action of *miR-196 *in this context (Fig. **4A**).

## THE CONTRIBUTION OF miRNAs TO A-P AXIS FORMATION

Striking phenotypes affecting A-P patterning have been observed following ectopic expression of Hox embedded miRNAs. In fly, either *miR-iab-4 or miR-iab-8* can induce a partial haltere to wing transformation, a classic manifestation of diminished *Ubx *[17,64,69]. Enforced *miR-10* in zebrafish causes a specific neural migration defect that can be rescued with the target gene *Hoxb1a *[18]. Finally, overexpression of *miR-196* in zebrafish induces a complete loss of pectoral fins (acting in this context through attenuation of retinoic acid signaling), and a reduction of the A-P axis [72]. For the most part however, overexpression phenotypes have not been consolidated with complementary loss-of-function phenotypes, suggesting that while these miRNAs have the ability to impact on Hox genetic hierarchies, their *in vivo* function is not apparent under wild-type conditions. These studies do not preclude a more subtle role for individual miRNAs in canalizing A-P axis formation in the fluctuating conditions of a natural environment. The one exception however is *miR-196*, which has an important, non-redundant, role in A-P patterning of multiple tissue types within the vertebrate embryo.

Vertebrate *miR-196 *is essential for regional segmental number and identity across a surprisingly large extent of the A-P axis (Fig. **4**) [14,16,72]. The most widespread and early knockdown of cumulative *miR-196* achieved to date is in zebrafish, and results in a dramatic overall expansion of the A-P axis [72]. Specifically, an increase in pharyngeal arch number was observed, however the molecular basis for this unexpectedly anterior defect is unknown and whether these defects are cell autonomous has not been tested. Additionally, a specific expansion in the number of rib-bearing precaudal vertebrae was identified [72] (discussed below). A rib-suppression activity for *miR-196 *was identified in chick, though likely achieved by different developmental mechanisms [16]. Additionally, *miR-196* clears Hoxb8 activity in the posterior neural tube where it would otherwise inhibit motor neuron differentiation [14]. In all experiments to date, the contribution of individual miRNA paralogs has not been clarified and remains an important area for future investigation.

These data support an emerging role for *miR-196 *in suppressing rib formation. Homeotic transformations observed at the C-T transition following *miR-196* knockdown in chick [16] are easier to rationalize in terms of ectopic Hox expression [77], though the exact spatial and temporal requirements for individual miR-target interactions requires careful analysis. How *miR-196* regulates somite number [72] remains to be defined and is particularly challenging to integrate into the current framework of Hox function determined over decades of mouse mutant analysis (it should be noted that dysregulation of Hox-target mRNAs in *miR-196 *morphants is yet to be demonstrated). Generation versus patterning of the A-P axis are largely considered separable genetically, with the former traditionally viewed as Hox independent though recent evidence is beginning to challenge this [78]. With only a few rare exceptions (e.g. *Hoxb-13*), axial defects observed following gain or loss of Hox function results in homeotic transformation (and/or malformations) not regionalized expansion. In this light however, it is particularly interestingly that early mesodermal deletion of Dicer in mouse causes a posteriorization of hindlimb positioning by 3 somites [79]. Whether this is accompanied by homeotic transformations or expansion of thoracic vertebral elements is not known since embryos die at mid-gestation [79]. While the molecular details still need to be worked out, the importance of *miR-196 *in shaping the A-P axis is beginning to be defined.

## A UNIFYING VIEW OF HOX EMBEDDED miRNAs

The Hox genes are under transcriptional and post-transcriptional regulation. While it is clear that *miR-196* regulation of Hox genetic networks is required for morphological outcomes along the A-P axis, this is not the case for all Hox-embedded miRNAs despite their molecular potential. This could reflect the often subtle nature of miRNA regulation, or possibly the active avoidance of miRNA regulation specifically at early developmental stages when the A-P axis is being established [80,81]. Future studies utilizing genetically or environmentally sensitized backgrounds may be required to uncover novel miRNA functions. 

The evolution and retention of multiple mechanisms to regulate the same suite of genes is conceptually intriguing. There are many instances whereby two mechanisms of different efficiency are required for the success of a complex biological system responding to environmental cues. The results of a recent study clearly describe the need for both high and low efficiency nutrient transporters in yeast to respond effectively to a naturally fluctuating environment [82]. This concept can be applied to gene regulation, by which two mechanisms of high and low efficiency, and therefore sensitivity to cellular cues, are required to maintain homeostasis. miRNAs may function as low efficient, more sensitive, regulators of gene expression; therefore, behaving as first responders to environmental fluctuations and relaying this information to more efficient, less sensitive transcriptional regulation.

Integral to the evolution and retention of novel morphologies is the ability of the underlying genetic networks to effectively respond to environmental changes and maintain homeostasis. miRNAs and their target sites are continually co-evolving in animals and in doing so, are predicted to impart greater developmental robustness, i.e. reduce phenotypic variability. miRNA control may be advantageous both in a shorter evolutionary time scale (limiting extensive plasticity in Hox expression that in many cases may have lethal patterning consequences), as well as being a molecular mechanism by which phenotypes are canalized and therefore visible by natural selection to drive the evolution of novel morphologies. The ancestral Hox expression patterns were possibly more broad and overlapping and by evolving an additional level of regulation over these expression domains, unique and reproducible A-P patterning could be achieved.
